# SUBJECTIVE COGNITIVE CONCERNS AND NEUROPSYCHOLOGICAL PERFORMANCE IN PRODROMAL PARKINSON’S DISEASE

**DOI:** 10.1093/geroni/igae098.4083

**Published:** 2024-12-31

**Authors:** Britney Luu, Katherine Bangen, Mary Ellen Garcia, Uriel Urias, Douglas Galasko, Kelsey Thomas

**Affiliations:** San Diego State University, San Diego, California, United States; University of California, San Diego, La Jolla, California, United States; University of California, San Diego, La Jolla, California, United States; San Diego State University, San Diego, California, United States; University of California, San Diego, La Jolla, California, United States; University of California, San Diego, La Jolla, California, United States

## Abstract

While subjective cognitive concerns (SCC) are commonly observed in preclinical Alzheimer’s disease, SCC in cognitively unimpaired individuals at risk for Parkinson’s disease (PD), as defined by reduced sense of smell (hyposmia) or REM behavior sleep disorder (RBD), is less understood. Therefore, this study examined associations between SCC and individual neuropsychological measures in the prodromal PD cohort (n=715, mean age=67.75) from the Parkinson’s Progression Markers Initiative (PPMI) study. SCC (n=199) or no SCC (n=516) was based on participant or caregiver report for item 1 on the Unified Parkinson’s Disease Rating Scale, part 1. Analyses of covariance, adjusting for age, education, sex/gender, race/ethnicity, psychiatric factors, motor symptoms, and prodromal classification (hyposmia or RBD), examined the association between SCC and measures of memory, language, visuoperception, processing speed, and executive functioning. Relative to participants without SCC, those with SCC had lower performance on Animal Fluency (B=-1.02, p=.009, np2=.011) and Symbol Digit Modalities (B=-1.68, p=.036, np2=.007). When examining the SCC x prodromal classification interaction, hyposmia or RBD status did not moderate the associations for Animal Fluency or Symbol Digit Modalities. Overall, SCC in prodromal PD participants was only associated with two measures requiring speeded retrieval and processing. Unlike in preclinical Alzheimer’s disease, where SCC is often linked to memory, no such association was found in prodromal PD. Instead, SCC was associated with speeded measures that are likely more sensitive to PD related changes. Future work will investigate the utility of SCC to predict conversion to PD and longitudinal rates of cognitive decline.

